# Area-level socioeconomic deprivation, non-national residency, and Covid-19 incidence

**DOI:** 10.1093/eurpub/ckac130.077

**Published:** 2022-10-25

**Authors:** S Rohleder, D Costa, K Bozorgmehr

**Affiliations:** 1 Department of General Practice and HSR, University Hospital Heidelberg, Heidelberg, Germany; 2 Department of Population Medicine and HSR, Bielefeld University, Bielefeld, Germany

## Abstract

**Introduction:**

Socioeconomic conditions affect the dynamics of the COVID-19 pandemic. We analysed the association between area-level socioeconomic deprivation, proportion of non-nationals, and Covid-19 incidence in Germany.

**Methods:**

Using nationally representative data at the level of 401 German districts from three waves of infection (January-2020 to May-2021), we fitted Bayesian spatiotemporal models to assess the association between socioeconomic deprivation, proportion of non-nationals, and Covid-19 incidence, controlling for age, sex, vaccination coverage, settlement structure, spatial and temporal effects. We estimated risk ratios (RR) and corresponding 95% credible intervals (95%-CrI) for deprivation quintiles. We further examined the deprivation domains (education, income, occupation), interactions between deprivation, sex and the proportion of non-nationals, and explored potential pathways from deprivation to Covid-19 incidence.

**Results:**

Covid-19 incidence risk was 15% higher (RR = 1.15, 95%-CrI=1.06-1.24) in areas with the highest deprivation quintile (Q5) compared to the least deprived areas (Q1). Medium-low (Q2), medium (Q3), and medium-high (Q4) deprived districts showed 5% (1.05, 0.98-1.13), 8% (1.08, 1.01-1.15), and 6% (1.00, 1.00-1.12) higher risk, respectively, compared to the least deprived. Districts with higher proportion of non-nationals showed higher risk compared to districts with lowest proportion, but the association weakened across the three waves. During the first wave, an inverse association was observed with highest risk in least deprived areas (Q1). Deprivation interacted with sex, but not credibly with the proportion of non-nationals.

**Conclusions:**

Socioeconomic deprivation and proportion of non-nationals are independently associated with Covid-19 incidence. Regional planning of non-pharmaceutical interventions and vaccination strategies would benefit from consideration of area-level deprivation and non-national residency.

**Key messages:**

- Area-level socioeconomic deprivation and proportion of non-nationals are independently associated with higher risk of Covid-19 incidence across 401 German districts.
- Tailored non-pharmaceutical interventions and immunisation strategies are needed, which properly consider area-level socioeconomic deprivation and non-national residents.

